# *“My job is to get pregnant women to the hospital”*: a qualitative study of the role of traditional birth attendants in the distribution of misoprostol to prevent post-partum haemorrhage in two provinces in Mozambique

**DOI:** 10.1186/s12978-018-0622-4

**Published:** 2018-10-16

**Authors:** Karen Hobday, Jennifer Hulme, Caroline Homer, Páscoa Zualo Wate, Suzanne Belton, Ndola Prata

**Affiliations:** 1 Menzies School of Health Research, Charles Darwin University, Darwin, Australia; 2 Department of Emergency Medicine, University Health Network, University of Toronto, Toronto, Canada; 3 Department of Family and Community Medicine, University of Toronto, Toronto, Canada; 4 Centre for Midwifery, Child and Family Health, Faculty of Health, University of Technology Sydney, Sydney, Australia; 5 Honorary Fellow, Menzies School of Health Research, Charles Darwin University, Darwin, Australia; 6 Maternal and Child Health Program, Burnet Institute, Melbourne, Australia; 7 Centre for Midwifery, Child and Family Health, Faculty of Health, University of Technology Sydney, Sydney, Australia; 8 Bixby Center for Population, Health and Sustainability, School of Public Health, University of California, Berkeley, USA

**Keywords:** Maternal health, Traditional birth attendant, Mozambique, Post-partum Haemorrhage, Misoprostol, Community

## Abstract

**Background:**

Post-partum haemorrhage is the leading cause of maternal deaths in Mozambique. In 2015, the Mozambican Ministry of Health launched the National Strategy for the Prevention of Post-Partum Haemorrhage at the Community Level. The strategy included the distribution of misoprostol to women in advance at antenatal care and via Traditional Birth Attendants who directly administer the medication. The study explores the role of Traditional Birth Attendants in the misoprostol program and the views of women who used misoprostol to prevent post-partum haemorrhage.

**Methods:**

This descriptive study collected data through in-depth interviews and focus group discussions. Traditional Birth Attendants between the ages of 30–70 and women of reproductive age participated in the study. Data was collected between June–October 2017 in Inhambane and Nampula Provinces. Line by line thematic analysis was used to interpret the data using Nvivo (v.11).

**Results:**

The majority of TBAs in the study were satisfied with their role in the misoprostol program and were motivated to work with the formal health system to encourage women to access facility based births. Women who used misoprostol were also satisfied with the medication and encouraged family and friends to access it when needed. Women in the community and Traditional Birth Attendants requested assistance with transportation to reach the health facility to avoid home births.

**Conclusions:**

This study contributes to the evidence base that Traditional Birth Attendants are an appropriate channel for the distribution of misoprostol for the prevention of post-partum haemorrhage at the community level. More support and resources are needed to ensure Traditional Birth Attendants can assist women to have safe births when they are unable to reach the health facility. A consistent supply of misoprostol is needed to ensure women at the community level receive this life saving medication.

## Plain English summary

Bleeding after childbirth (post-partum haemorrhage) is the leading cause of maternal deaths in Mozambique. The majority of these deaths happen when women give birth at home, without a skilled or trained health worker. In 2011, the World Health Organization stated that they support the distribution of misoprostol (oral medicine tablets) to prevent bleeding after childbirth. These tablets are important for women who cannot get to a health clinic to give birth because they are easy to use, prevent bleeding and can save their lives. A woman simply swallows three tablets of misoprostol directly after the baby is born and before the afterbirth (placenta) is delivered.

In 2015, the Mozambique Ministry of Health launched a National Strategy to prevent bleeding after childbirth for women who give birth at home. The strategy included two ways for women to receive the misoprostol tablets. First, women can get misoprostol when they visit the health clinic for a maternal health check-up at 28 weeks in their pregnancy. Or second, they can receive it from a Traditional Birth Attendant, a community woman who helps other women give birth. Traditional Birth Attendants receive training about the medication and how to assist women in childbirth but is not a qualified health worker. The Traditional Birth Attendant gives the misoprostol to the woman directly after the baby is born. The study looks at the role of Traditional Birth Attendants in the misoprostol program and the views of women who used misoprostol to prevent bleeding after childbirth.

## Background

The maternal mortality ratio in Mozambique is 489/100000 live births [1] and post partum haemorrhage (PPH) is a leading cause of maternal deaths [2, 3]. Mozambique experienced a 64.8% reduction in maternal mortality between 1990 and 2013 [1, 4] almost reaching the Millennium Development Goal target of a 75% reduction. Still, the lifetime risk of maternal death remains high; one in 40 Mozambican women will die during child birth [1]. The Mozambican Health Sector Strategy 2014–2019 prioritises the reduction of maternal mortality as a primary goal [2].

Results from a 2015 survey in Mozambique found that overall, 70% of births were assisted by Skilled Birth Attendants (SBA) [5]. The Ministry of Health marked significant improvement from the Demographic and Health Survey (DHS) in 2011 which found SBA coverage to be only 54% [1]. However, disparities exist; disaggregated results from urban and rural populations show heath facility births are much more prevalent in urban areas (91%) compared to rural areas (67%) [5]. Access to rural health services in Mozambique remains limited by geographic distance, lack of transport options, coverage and quality [6, 7].

In Sub-Saharan Africa, approximately 23% of all births are attended by a Traditional Birth Attendant (TBA, only half of which were formally trained in modern medical childbirth techniques with a focus on clean delivery [8]. A TBA is defined as, ‘community-based providers of care during pregnancy, childbirth and the postnatal period’ who are traditional, not-formally trained and work independent of the health system [9]. TBAs were largely ignored through the 1990s by governments and the global health community after multiple studies suggested that TBA training was cost ineffective and had minimal impact [10–12]. Notably, TBAs were not armed with birth kits or uterotonics alongside this training and thus some argue that new technology and evidence could improve outcomes [12, 13]. There is also growing evidence that TBA training may improve linkages with facilities and improve perinatal outcomes [10]. The aim of this paper is to analyse the role of TBAs in the provision of a maternal health innovation in Mozambique.

## Context in Mozambique

Home births are a reality for women in rural and remote areas of Mozambique, despite the country’s policy to achieve the highest possible coverage of facility births with SBAs. TBAs play an important role in promoting ANC, often accompany and attend facility births, and assist at home births when women cannot access the facility births. Women in rural Mozambique often give birth at home with a TBA who may have limited or no training and few resources to ensure a safe delivery.

Prior to 1985, the MoH largely discouraged the involvement of TBAs as they were seen to detract from facility based births [14]. Investment and training in TBAs commenced in the late 1980s in Mozambique as an acknowledgement of the shortage of SBAs and limited coverage of facility births amongst rural women. TBA training first took place between 1991 and 1998; 3734 TBAs were trained, one-third of which were located in Zambezia Province [14, 15]. In 1996, a study of TBA training in Manica Province found no significant difference in maternal, perinatal or infant mortality between trained and untrained TBAs [14]. This was published amongst mixed evidence on the utility of training TBAs for improving the quality of care and reducing the mortality of mothers/newborns [10–12]. The authors recommended that TBA training be balanced with improvements to health facility infrastructure and the provision of professional support to midwives working in remote locations. Since 2014, there has been a concerted effort in Mozambique to re-engage in promoting early antenatal care (ANC) and facility births [2]; they have also engaged TBAs in the use of both chlorohexidine for the prevention of neonatal sepsis and misoprostol for the prevention of PPH [16].

Misoprostol has proven to be a safe alternative uterotonic where oxytocin is not available, such as at home births [17, 18]. Numerous countries have piloted the community-based distribution of misoprostol using either advanced distribution for self-administration during ANC visits or via Community Health Workers (CHW), or through distribution to TBAs who attend births [19–24]. Scale-up of community-based distribution of misoprostol remains limited; while there are early success stories emerging from Nepal and Bangladesh, many programs have been delayed or abandoned by donors or governments for political reasons and the lack of trust in community distribution [25].

The Mozambique MoH has committed to the expansion of misoprostol for the prevention of PPH alongside a network of CHWs known as Agentes Polivalentes Elementares (APEs) [15] and newly trained TBAs. A pilot distribution of misoprostol conducted in Nampula, Mozambique from November 2009–October 2010 found that distribution through both TBAs and Antenatal Care (ANC) achieved 99% misoprostol usage at home births [26]. In 2011, the Mozambican MoH formally approved the distribution of misoprostol at the community level for the prevention of PPH.

In 2014, the MoH launched the ‘Strategy for the Prevention of PPH at the Community Level’ (referred to as the National PPH Strategy), which included guidelines for the use of misoprostol for PPH where SBAs are not available [16]. The aim of the misoprostol program is to increase access to misoprostol for women who give birth in the community to reduce maternal mortality associated with PPH. The National PPH Strategy named 35 districts across the country to be included in the program for distribution. TBAs have a key role in the distribution and administration of misoprostol for the prevention of PPH. Pregnant woman at 28 or greater weeks of gestation may receive misoprostol in advance at ANC or they may receive it via a TBA in their neighbourhood. APEs act as the link between the health facility and the TBAs by providing misoprostol to the TBA in their zone. APEs are formally recognised by the health system, have a higher level of education than TBAs, often have a bicycle for transport, and receive a monthly subsidy [27]. Misoprostol for the prevention of PPH is branded as ‘Misol’ as a way to differentiate it between misoprostol used for inductions or abortion.

Implementation commenced in 2015 in six districts in Inhambane and Sofala provinces, and is ongoing in 35 districts across the country at the time this article was sent to publication. TBAs were recruited via recommendations by the health staff or APEs. Distance from the health facility was not considered in recruitment. Some TBAs prefer to remain autonomous and maintain their status outside of the formal health sector. Health staff, TBAs and APEs all received training on the use and distribution of misoprostol in May 2015 in Inhambane Province and August 2016 in Nampula. TBAs received three- day training on safe birth practices and the use of misoprostol; 47 TBAs were initially trained in Inhambane and 80 in Nampula Province. Training was provided in a cascade ‘Train the Trainers’ approach and was supported by UNFPA and Jhpiego’s Maternal and Child Survival Project. TBAs distribute misoprostol to women directly at the birth and are not permitted to provide the misoprostol in advance.

The aim of this study was to explore the role and perceptions of TBAs in the distribution of misoprostol for the prevention of PPH at the community level in two provinces in Mozambique. The facilitators and barriers that TBAs experienced working within the misoprostol program were also examined. We also sought to understand the views of women who had used misoprostol for the prevention of PPH.

## Methods

### Study design

This research was nested within a larger implementation study evaluating the distribution of misoprostol for the prevention of PPH in two provinces in Mozambique. A phenomenological qualitative approach was applied to understand the misoprostol program through the experience of TBAs and women who had used the medication. This approach is suited to understanding health care and health systems through the real world experiences of patients and health workers engaged in the system [28]. A combination of focus group and individual interviews allowed a broad range of themes, which were then further explored in focus group discussions. It also allowed inclusion of a more diverse group of participants whose availability and comfort with the two methods might differ.

This study was descriptive and used semi-structured qualitative interview guides for data collection. In-depth interviews and focus group discussions (FGD) were conducted by a trained research team who consisted of both local and international research assistants. Semi-structured interview guides ensured the main topics were covered including use and understanding of misoprostol, supply, safety, barriers and facilitators to participation in the program. A narrative review of the evidence base, challenges and scale-up of misoprostol was conducted by the authors [25]. This review in part informed the topics in the interview guide. The interview guides were pilot tested and then revised to ensure the questions were clear.

The majority of interviews and FGDs were conducted in local language or where appropriate, Portuguese. Interviews were held between 30 and 60 min and were recorded with permission from the participants. At the end of each day, individuals and/or the team debriefed their observations and provided further details about the interviews. Ethical clearance was granted by the Human Research Ethics Committee at Charles Darwin University, Australia (HREC 2015–2445) and the Mozambican National Bioethics Committee and Ministry of Health. Informed consent was sought and gained for all participants.

### Setting

The study took place in three districts in Nampula Province and two districts in Inhambane Province in Mozambique. Inhambane and Nampula Provinces were chosen due to geographic region; one study site was located in the Southern region and one in the Northern Region of the country. Districts in each province were chosen based on inclusion in the national misoprostol for prevention of PPH program, geographical location and on discussions with Provincial and District Health Authorities.

### Study participants

Female TBAs between the ages of 30–70 were the key participants in the study. Respondents were selected based on geographical location, involvement in the misoprostol program and selected with assistance from either the health facility staff and/or the CHW. Where possible, a research assistant would call the TBA directly to request the interview. In some cases, the District Maternal Health Director and/or Community Health Workers were asked to contact the TBAs in their designated district and invite them to the health facility to speak to the study team. Participants were given the option of withdrawing from the interview or the study at any point; no participants requested to withdraw. Additionally, interviews were conducted with women of reproductive health age in Inhambane and Nampula Provinces who had previously used misoprostol to prevent PPH during childbirth. These women were purposively selected on recommendation by the TBA, using snowball sampling to access this hard to reach population. Participant numbers were determined based on obtaining thematic saturation. Data were collected between June–October 2017.

### Analysis

All interviews and debriefs were translated and transcribed in Portuguese, and then translated into English. Line by line thematic analysis was used to code and analyse the data using NVivo (v.11) [29]. KH and JH initially read all interviews twice to familiarise themselves with the data. Interviews were coded initially based on emerging themes, and then re-read and coded again resulting in additional themes. KH and JH coded the interviews and FGDs independently and reached consensus on key themes after several discussions; they discussed divergent cases as they arose and co-authors provided input when necessary [30]. Notes from debriefings and recordings were reviewed and informed the analysis, particularly as a reminder of the context. KH is undertaking her PhD and JH is a medical doctor. They both have research and programming expertise in public health. Their views and life experiences may have influenced the data analysis.

## Results

Qualitative semi-structured interviews were conducted with 16 purposefully selected Traditional Birth Attendants (TBA) (11 in Inhambane and 5 in Nampula Provinces). Four FGDs were undertaken with TBAs in Nampula Province with 3–5 women in each group. In total, we interviewed 11 women who had used misoprostol; 5 women were interviewed individually in Inhambane and Nampula Provinces and 6 women participated in a FGD in Nampula Province.

**Fig. 1:**
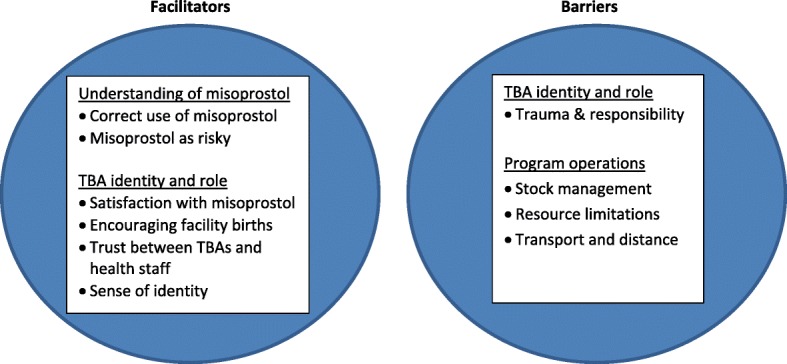
Key Themes and Sub-themes

The findings were organized in the following themes: a)Understanding of misoprostol: correct use of misoprostol; misoprostol as risky b) TBA identity and role: satisfaction with misoprostol; encouraging facility births; trust between TBAs and health staff; sense of identity;, trauma and responsibility and c) Program operations: stock management;, resources limitations; and transport and distance. These themes were then organised in the context of facilitators (groups a and b) or barriers (group c and trauma and responsibility) to the misoprostol for prevention of PPH program. See Fig. 1.

### Understanding of misoprostol

#### Correct use of misoprostol

The vast majority of TBAs correctly understood how to administer the misoprostol: three pills (600 micrograms) after the woman had given birth and prior to the removal of the placenta. For example, “*The mother, after giving birth, and before the placenta [is birthed], has to take three pills before the placenta leaves to reduce postpartum haemorrhage” (TBA1, FGD 2).* Another TBA explained, *“In the training I was informed as soon as a mother finishes giving birth, I must immediately give three pills and she should swallow immediately to avoid bleeding” (TBA 4).*

Three TBAs were unsure of how to administer the medication and they had not had the misoprostol provided to them since receiving the training. Because training took place in May 2015 in Inhambane Province and August 2016 in Nampula, there was often a time lag between training and the actual distribution of misoprostol. The majority of TBAs clearly described the administration of misoprostol prior to birth as ‘dangerous’ and knew the exact timing to give the medication. When asked if there were any other uses for misoprostol, none of them could name any, including when probed about use for abortion. However they were strictly told during their training to hide the medication from children and for others, saying for example:

*Uhhhh, I personally have never heard of it [giving misoprostol before the birth], see even in the training about Misol we were not told that, we were told to give it after the birth, before the birth it should not be given. (TBA 9)*

### Misoprostol as risky

There was a strong focus on the risks and dangers (both real and fictional) associated with misoprostol as taught during the training and reinforced by the nurses. The TBAs receive three doses in a blister pack from the APE. If a zone or neighbourhood did not have an APE, the TBA received the doses directly from the Maternal Newborn and Child Health (MNCH) nurse at the health facility. Many TBAs were told that the medication, despite being in closed blister packs, attracted rodents and there would be a high risk of death if consumed by a child. One TBA explained,

*They told us that we should be very careful with these tablets, keep them very well and safely, if they are opened even if it is a small opening, they become unusable, so we should not administer in this situation. (TBA 13)*

One TBA mentioned that if the drug were to be sold, that the TBAs would experience severe consequences. “*…we were warned not to sell any pills, anyone found to do this could pay a fine of one million and go right to jail” (TBA 14).*

### TBA identity and role

#### Satisfaction with distribution of misoprostol

Overall, the majority of TBAs stated that they had positive feelings about administering misoprostol and believed it was useful for their work and the community. They made statements such as:

*... the pills really help a lot, because there are times when the patient loses a lot of blood and if there are no pills it can be fatal, especially since everything here is distant, there is no transport and in the middle of the bush little can be done to save the patient. (TBA 15)*

*When we got the child out, I took out the Misol and gave it to the lady and removed the placenta. There was no bleeding problem. I am very pleased with this work. It is rewarding when everything goes well without bleeding, and even if the delivery goes well, I always advise her to go to a hospital for a brief check-up. (TBA3, FGD 3)*

Women in the community who had taken misoprostol also spoke positively about the medication and felt that it made them strong and their ‘body firm’. The majority of the women did not know the name of the medication; they referred to it as the pill that stopped the bleeding. All stated that they wanted the program to continue, for instance:

*When I left the hospital I walked by myself until I got home. I did not need to stay in bed or be hospitalized, the pill had a good effect on my body. I was able to walk by myself. (Participant 3, FGD 6 Women who used misoprostol)*

A few women spoke about some people who had not yet taken misoprostol that had worries about taking medication. They said that they encouraged other women in their community to use misoprostol as this woman explained.

*People who have not yet taken it [misoprostol] say they are afraid to take it because they do not know what the reaction will be. Now we have tried to pass on to them the experience that it is a good medicine and that it does not harm the body and that it does not kill, we are here alive and healthy. (Participant 2, FGD 6 Women who used misoprostol).*

*The men when their wives have not taken it yet say that this pill is nothing, people are just excited, but for those of us who have already taken it our husbands have a contrary opinion because they have already seen the good effects of the Misol. (Participant 1, FGD 6 Women who used misoprostol).*

#### Encouraging facility based births

All TBAs in both Inhambane and Nampula provinces emphasised that their primary responsibility was to bring women to the health facility to give birth. TBAs sometimes give talks to the community and women about seeking antenatal care, giving birth with an SBA and childhood vaccinations. APEs and community leaders also play a role to encourage health seeking behaviour and facility births. Women who used misoprostol also said that they encourage friends and relatives to seek care early and, if needed, use misoprostol in cases when they cannot get to the facility, for example:

*…since the government has thought of putting a traditional midwife [TBA] in each neighbourhood or community, several advances have been recorded. In fact, it was a wise decision. And the mobilization or campaigns of sensitization is not only done here in the hospital, but also in the neighbourhoods. Local structures such as Ward Clerks, Community Leaders, are all aligned on spreading information about pregnancy and Misol. Even the structures of the district government have collaborated. When we met with a Director and asked for help to take a woman to the hospital, there was never anyone who refused. We all collaborate. (TBA 3, FGD 4)*

It was commonly accepted that misoprostol was to be used only if a woman could not reach the health facility. Most of the informants, unprompted, described the importance of facility births as the safest option. TBAs described misoprostol as a medication only to be used in the event of a ‘surprise’ birth, or ‘birthing along the way’ to the facility. Any birth that they assisted with at home was either because the woman would give birth on the road, had progressed too quickly, could not walk, or a transfer could not be arranged despite her efforts. TBAs made comments explaining this including:

*When I started this program, we were taught that we cannot perform births at home, but there may be some surprises and we have to deliver before we get to the hospital, so we should have these pills to give the woman after the baby is birthed. However, it is our duty to mobilize and sensitize pregnant women to the health unit. (TBA 6)*

Some TBAs took a staunch approach to implementing the policy on health facility births and refused to assist home births. One TBA mentioned that ‘their house was not a maternity’ and they would outright refuse women to enter their house to give birth there, for example:

*No, I do not usually take much time, when the place is far away I refuse (laughs), because I teach them to go to the hospital, going to the patient’s house is also not accepted, only to call me, saying that she is on her way to the hospital. I do not accept to receive people in my house to give birth; they did not teach me to transform my house into maternity (laughs). (TBA 15)*

#### Trust between TBA and health staff

Trust was identified as a major theme in the study and was a facilitator to the program being accessed in the community. Trust was central to allowing the health facility to move forward with community distribution. TBAs are well known in their community and respected which assisted in introducing misoprostol as a new intervention. Most TBAs enjoyed a positive relationship with those involved in the misoprostol program including the nurses, health staff and APEs.

TBAs often worked closely with the APE in their neighbourhood. They mostly described a good working relationship with the APE and were satisfied with the role the APEs played as the channel between themselves and the health facility for the distribution of misoprostol. For example,

“*We take good care of them, along with our nurses, they do not abandon us nor despise us, in fact you just see between us and the nurses we have a good relationship…we understand each other” (TBA 7).*

A strong, positive relationship with health staff also appeared to anchor the program and encourage facility births. Many TBAs spoke about attending the labour after assisting the woman to reach the health facility, some TBAs said they would observe or assist in the maternity ward. All TBAs appreciated and felt respected when the nurses included them in the maternity ward. Others mentioned that it was a good opportunity to learn from skilled birth attendants, as illustrated here:

*We have good relations, we talk, we respect [each other], we help ourselves and we teach a little of everything, so that the service can go well, after all we all have the same objectives …they always receive [us] with great professionalism, both the traditional midwives [TBAs] as well as the patients that we bring from our communities. Even when we arrive at midnight, the nurses get up and greet us. (TBA 1, FGD 4)*

However, not all TBAs reported a positive relationship with the nurses and some were unhappy with the dynamic, for example:

*That's why I said that when I arrive at the hospital with a patient, the nurses do not give me importance, until at some point they ask where you are going to sleep? They do not give me the opportunity to watch the work with the more informed colleagues, which is not good. (TBA 4)*

#### Sense of identity

TBAs very strongly identified with their role, some stated that their skills to assist with births were a gift from God, or a skill passed on by their mother or family member. Often they were chosen by the community to work as a TBA.

*I used to do many births, it was not by accident that I was taken to work in coordination with the hospital, although I could not read or write, but God gave me this gift to help women. Nowadays, since we started talking about the need for women to go to the hospital, these cases have greatly diminished. (TBA 8)*

TBAs mentioned the importance of having t-shirts or capulanas (traditional material worn as a skirt, dress or to carry babies) with MoH logos to help with their identification in the community and to help them to gain acceptance and be respected in their work. The TBAs that had received a MoH printed TBA t-shirt wore it very proudly; it was clear this small incentive was a positive means to show they received support from the MoH. For example:

*As we have this t-shirt, although it is the only one that was given to us in the hospital [a TBA printed MISAU shirt], it helps them to identify us and see that we work in coordination with the hospital and so they do not reject us. (TBA 7)*

#### Trauma and responsibility

Very few TBAs experienced maternal mortality or newborn deaths, and several noted that they experienced fewer cases of PPH after the introduction of misoprostol. Those that had experienced deaths described a deep sense of responsibility despite their limited skills and resources. This is highlighted in this quote:

*There was this one time when I lost a girl here in the house, it was very difficult. Another time as well there was a woman whose family I counsel to go to the hospital but they took their time, and when they arrived at the maternity unfortunately it was too late, and she died. (TBA 10)*

They also described significant fear and anxiety around attending homebirths without having the skills or the materials to respond adequately, for instance:

*What usually is difficult for me is when you advise someone to go to the hospital and they resist until they give birth at home, it makes me very sad, because there may be a serious problem with the baby or the mother and I am held responsible for being the TBA of that person… Imagine that she gives birth and needs stitches, how will I do it? So I should be able to make the woman come to the hospital on time. (TBA 12)*

Overall TBAs felt very accountable to their community and also worried about being blamed for any adverse outcomes. They had a clear sense of responsibility to respond to the needs of pregnant women and women in labour. This also manifested in fear and guilt, that they will not be able to provide for women when needed as explained here:

*No, because as soon as you said that you are looking for the pills, for now there are none. This would be the only request because if you find someone in labour, outside the hospital, it is difficult without the pills, and we can only trust God, since we can not leave the person suffering. (TBA 16)*

### Program operations

#### Stock management

Stock outs were a source of stress and concern for both TBAs and women; there was concern for the women who would not benefit from the misoprostol. In the National PPH Strategy, the process for distribution of misoprostol is from the Health Facility to the APE to the TBA. TBAs receive the misoprostol in a blister pack from the APE. The APE acts as the link between the health facility and the TBA as they often have bicycles and a formal connection to the health system. APEs are responsible to submit monthly reports to the health facility and can then pick up more stock of misoprostol if needed. In practice, this system does not always function. In some instances, TBAs were not able to retrieve the misoprostol from their identified APE due to distance, unavailability or illness. In other cases, there may not have been an APE who lived in the same neighbourhood or community as the TBA. Some TBAs bypassed the APE “middle men” and picked up the misoprostol directly from the health facility. For example, “*They told us in the training that it should be the APE to give us but for my case it is difficult because my APE is far from me, so I come here to pick it up at the health unit with the nurse” (TBA 5).*

Other TBAs mentioned that the system whereby the TBA received the misoprostol from the APE was not always functioning due to the distances between the APE and TBA and the reliance on cell phones for communication. One TBA explained it as the following, “*We work well [together], but I’m very far from the APE, only my colleagues are close to them. I have more contact with the nurses” (TBA 13).*

In some districts, TBAs had been without stock for several months and in a few cases up to a year. Other TBAs said they had received training, but had not yet received any doses of misoprostol. This raised questions about the sustainability of the program; TBAs without regular access to misoprostol questioned whether the program would continue. In other cases TBAs reported that they had received stock but had to return it as the pills were expiring, for example:

*When they do not have it we have a real despair. As it is a medicine that helps we run to the hospital to ask the nurse or the midwife. The sad thing is when they say they do not have it and are waiting for it. But when it's in the warehouse there's been no difficulty in giving us the pills. (Participant 2, FGD 6 Women who used misoprostol)*

Surprisingly we did not speak to any TBAs that supervised the administration of misoprostol that mothers had previously received at ANC. TBAs who lived further away from the facilities were often those who directly administered misoprostol, probably to women who were less likely to have had received ANC after 28 weeks. A reliable supply of misoprostol which is easy to access, either via the APE or directly from the health facility was seen to be an essential component of the functioning of the program.

#### Resource limitations

Lack of financial and material resources were prominent underlying barriers to the TBAs involvement in the misoprostol program. TBAs do not receive any salary, incentives or resources for their participation in the program. While this did not seem to have negative implications on the relationships between the TBA and APEs or health staff, all of the TBAs mentioned that they would appreciate support from the government to perform their jobs safely. TBAs that we spoke with did not have any other employment or additional income and were very poor, for example:

*Unfortunately I am not lucky enough to earn money for the work I do, I have already brought many people into the world by my hands. If it were a case of working for a boss, I think I would be in retirement now and earning money for the time spent in this activity. (TBA 15)*

Many TBAs spoke about their lack of soap and gloves, and resulting fear of infection. They explained that they did not receive gloves or soap to assist with their work as their role, as per MoH policy, was solely to accompany woman to give birth at the health facility, as explained here:

*Well, they tell me that my job is to get pregnant women to the hospital, but if it happened before I was there, I should get some plastic [from a 1kg sugar bag], and put it on my hands, that's how I do it, they did not give me gloves, they said they wear the gloves in the hospital. (TBA 10)*

*Just a few things saddens me in this work, we do not want much, just soap, imagine you my daughter, we are dirty and we have to go home to wash us with the little that our husband tries to arrange, with many difficulties, he even gets annoyed because he says that this work does not help me at all and he threatens to forbid me. Still, I keep doing it because I like to help. Therefore, we do not need much, just soap because it is inevitable that the blood will make us dirty and it is not easy without cleaning ourselves. (TBA 7)*

Poverty and shame was also an underlying theme in discussions with women who had used misoprostol. Women spoke of poverty in terms of the lack of resources to deliver comfortably; being transported to the health facility, with a new capulana (traditional cloth) for the baby, and having food to eat after the birth of the baby, for example:

*For example when a child is born they do not let it be covered with this capulana that I used, in the hospital they say it must be a new capulana ... where will I get money for a new capulana if my only capulana is this one? So we do not know ... this price we are paying for our misfortune is very high. The nurses only know to say that we should produce vegetables to be able to sell to get money ... but here who will buy what we produce if we are all farmers and poor people? (Participant 3, FGD 6 Women who used misoprostol)*

#### Transportation and distance

In addition to a lack of resources, the majority of TBAs spoke about the challenges they faced reaching the health facility. One of the key roles of TBAs is to accompany women to the health facility to give birth; however transportation was never provided, and many TBAs reported they had to walk or pay for transport out of pocket as highlighted here:

*Another problem is transportation, I live far from the health unit, I must pick up two cars and pay 40 meticais just to come, so, round trip is 80 meticais and no one gives me that money, its personal effort. We are told that this is voluntary work and that we should do it of our own free will, so I do it without gaining anything. (TBA 13)*

A few TBAs refused to assist in home births, even when transport or walking was impossible, and seemed concerned about being blamed or punished by heath staff if and when they did. ‘Birthing along the way’ or on the side of the road in transit to the health facility was a challenge for both TBAs and women. TBAs described the challenges of walking and assisting women to give birth in the dark without gloves, soap or water. TBAs and women explained that often they only had an unwashed capulana (traditional cloth) with them to wrap the baby.

Women in the community vividly described the hardship of walking 3 hours or more to the health facility in late pregnancy or in labour. The imperative for safe reliable transport to the health facility was a key request by women in the community:

*We want to continue to receive your medicines the Misol tablet. Of the many difficulties that women here face the greatest of all is transportation. We need a car to help us. Our bodies are already getting tired, we have to raise children, take care of the house and still go to the fields. We are asking for a lot [a car]... with this, expensive Misol would reach the most distant people... (Participant 5, FGD 6 Women who used misoprostol)*

## Discussion

This study aimed to understand the role and perceptions of TBAs in the distribution of misoprostol for the prevention of PPH at the community level in two provinces in Mozambique. The facilitators and barriers that TBAs experienced working within the misoprostol program were also examined.

We encountered tremendous support for the misoprostol program from TBAs and women themselves. Women who had used misoprostol appreciated the medication and encouraged community members, relatives and friends to use it. TBAs highly valued the program, and spoke confidently about distributing misoprostol and understood its role in preventing PPH. Similar to other studies in Nigeria [31] and Nepal [32], most TBAs correctly explained how and when to administer misoprostol.

TBAs expressed a strong sense of identify and pride in their work, and described their role as a respected member in their community. Many TBAs spoke of the calling they had either from God or a family member to assist women to give birth. T-shirts and capulanas with the MoH logo provided them with a uniform and a means of identification in the community. TBAs felt a heavy responsibility to the women they assist which at times caused them to feel stress. Few TBAs were present when a woman passed away in labour or shortly after giving birth; however many discussed the risk of death and their fear of being able to assist with complications.

TBAs described strong, positive relationships with the APEs and MNCH nurses which often bolstered their sense of identity as a link between the informal and formal heath system; other studies have also seen an increase in referrals and awareness of medical care with the engagement of TBAs [33, 34]. TBAs were satisfied that their work was recognized by the formal health sector, and interestingly some appreciated the opportunity to observe the facility delivery as a learning tool. These findings are in keeping with a systematic review which found that including TBAs within health facilities increases skilled birth attendance and the more involved the TBA was with the health facility, the greater the impact [35]. Facility births can be improved with the creation of a supportive environment that links TBAs and SBAs and also removes barriers to women’s access to health facilities and SBAs [32].

TBAs consistently described accompanying pregnant and labouring women to the health facility, a finding in keeping with the strong commitment by informal and formal health care workers and community members in southern Mozambique to promote ANC and births at the health facility [7]. TBAs were aware of the national policy to encourage health facility births, and understood that misoprostol should be used only in the event of emergencies or unexpected births. Findings from a recent systematic review of task shifting in Active Management of the Third Stage of Labour support this approach; first promoting facility birth and then including additional interventions, such as community distribution of misoprostol, in place as a safeguard [36]. While it is encouraging that misoprostol is being used only as a last resort, it is important also that health providers and TBAs receive the message that it is a life saving medication and therefore they should not be overly cautious and avoid using it for fear of repercussions.

This study unveiled several unintended consequences of promoting facility deliveries without major investments in transport or communication systems. While some communities did have access to a maternal waiting home, often women were not able to leave their children at home without care and/or husbands did not see it as appropriate for their wife to be away from the home. Giving birth “on the way” to the facility, with no protection from the elements has also been described in Malawi, where women had to give birth on the roadside often due to a delay to seek assistance from a TBA or SBA at the health facility [37].

Some informants attributed their lack of resources for safe and clean birth as a way the health facility emphasised their role to promote facility births. Almost all TBAs discussed the lack of soap and gloves as having a negative impact to their work and motivation. Some TBAs spoke about using alternatives like used sugar bags or plastic sheets as makeshift gloves or none at all. They were fearful of contracting blood-borne illnesses themselves. Clean birth kits are not available to the TBAs; this includes soap, gloves, plastic sheets, razor blade and a cord clamp or string [38]. Clean birth kits are an affordable intervention and relatively easy to implement; they are used in at least 51 low-income countries with high prevalence of home births [38]. Lack of gloves and soap was seen to be a greater barrier to the work of TBAs than any other, including transportation and lack of financial incentives.

Poverty was the root of discussions around TBA and women’s lack resources, and transportation. TBAs do not receive payment for services besides what the community or households may offer informally – food, material or other small gifts. APEs are supported by the national program and receive a monthly subsidy of approximately 20 USD. Despite not receiving a stipend or incentives, the majority of TBAs said that they did not feel resentment towards the APEs for their earnings or expect a salary. However, many were impoverished by the out of pocket transportation costs associated with accompanying women to the health facility. All TBAs in the study said they would appreciate basic resources to assist with their work. Many described the hardship of encouraging facility based births coupled with the reality of the great distances, lack of transportation, lack of money and poor access. These barriers to healthcare are widespread across Mozambique as cited in other studies [6, 7, 39].

This study supports the notion that TBAs can be effective partners in delivering maternal and child health interventions to improve safe delivery until health facility birth is accessible to all women [10, 13]. The results of this study provide insight into the role and daily work of TBAs in Mozambique, with implications for minor modifications to the operations of the national misoprostol program, and implications for maternal health policy more broadly. Recommendations in Table 1 reflect a combination of global best practices and conclusions from this study.

**Table 1:** Recommendations

Operational recommendations for the misoprostol program:	
• Allow TBAs to pick up the medication from the health facility where necessary and appropriate to ensure a stable supply of misoprostol.	
• Heighten communication of the Strategy for the Prevention of PPH at the Community Level to TBAs. TBAs and MNCH nurses should feel confident distributing and administering misoprostol to women who will have a home birth as part of the National Strategy.	
• Provide clear information to the community via heath facility staff, CHWs and TBAs about misoprostol and how to use it correctly, alongside messages encouraging facility-based birth to dispel fears and myths in the community.	
• Consider clean birth kits distributed through ANC, APEs, and/or directly to TBAs. This would alleviate the concerns TBAs have about infections to themselves, women and newborns while reducing neonatal mortality. This study found no concerns that this might undermine facility deliveries.	
Recommendations to increase coverage of births attended by SBAs:	
• Transportation, while costly, is a necessary investment to ensure women have access to health facilities. Women and TBAs both strongly support facility deliveries, but requested assistance with transport and communication.	

### Limitations

Participant recruitment was established through purposive sampling. A mapping of all TBAs in the country has not been conducted and very little reporting takes place due to a number of factors, including illiteracy and the fact that TBAs are not officially part of the formal health sector. Therefore it is very difficult to know details about the number of TBAs and their catchment areas.

The majority of TBAs that participated in the study and were involved in the misoprostol program live relatively near to the health facility. Therefore there is a risk of potential selection bias as TBAs were referred by the health facility or CHWs. Due to time limitations and participant availability we were only able to reach a small sample of women who had previously used misoprostol. The perspectives from TBAs who live further from the health facility are different than those who live relatively closer, especially in terms of access to the health facility and participation in the misoprostol program.

As with many qualitative studies, it is difficult to generalise the findings of this study due to the small sample size and varied context.

## Conclusion

This paper contributes to the growing body of research supporting the community distribution of misoprostol for PPH prevention in settings where births that take place outside of health facility are still a significant proportion of all deliveries, and provides particular contextual findings for the role of TBAs in two provinces of Mozambique. TBAs play an important role in the distribution of misoprostol, encouraging facility based birth and advocating key messages for safe motherhood. This study adds to the evidence that TBAs are appropriate channels for the distribution of misoprostol for prevention of PPH; they are willing, capable and understand how and when to safely distribute the medication. Many TBAs highly valued their inclusion in the health system, receiving training and being provided with misoprostol.

A consistent supply chain of misoprostol is necessary to ensure the sustainability of the program. TBAs request resources such as birth kits, communication and transport options to heighten their efforts to ensure women can access to the safest birth possible. The MoH should expand their support to TBAs as allies with the health system to improve maternal and child health outcomes.

## Abbreviations

ANC: Antenatal care
APE: Agentes polivalentes elementares
CHW: Community health worker
FGD: Focus group discussion
MNCH: Maternal newborn and child health
MoH: Ministry of Health
PPH: Post-partum haemorrhage
SBA: Skilled birth attendant
TBA: Traditional birth attendant
